# The Drug-Resistance Mechanisms of Five Platinum-Based Antitumor Agents

**DOI:** 10.3389/fphar.2020.00343

**Published:** 2020-03-20

**Authors:** Jiabei Zhou, Yu Kang, Lu Chen, Hua Wang, Junqing Liu, Su Zeng, Lushan Yu

**Affiliations:** ^1^Institute of Drug Metabolism and Pharmaceutical Analysis, College of Pharmaceutical Sciences, Zhejiang University, Hangzhou, China; ^2^Department of Urology, Cancer Hospital of Zhejiang Province, Hangzhou, China; ^3^The First Affiliated Hospital, School of Medicine, Zhejiang University, Hangzhou, China

**Keywords:** platinum-based anticancer drugs, transporter, apoptosis, autophagy, DNA repair, resistance

## Abstract

Platinum-based anticancer drugs, including cisplatin, carboplatin, oxaliplatin, nedaplatin, and lobaplatin, are heavily applied in chemotherapy regimens. However, the intrinsic or acquired resistance severely limit the clinical application of platinum-based treatment. The underlying mechanisms are incredibly complicated. Multiple transporters participate in the active transport of platinum-based antitumor agents, and the altered expression level, localization, or activity may severely decrease the cellular platinum accumulation. Detoxification components, which are commonly increasing in resistant tumor cells, can efficiently bind to platinum agents and prevent the formation of platinum–DNA adducts, but the adducts production is the determinant step for the cytotoxicity of platinum-based antitumor agents. Even if adequate adducts have formed, tumor cells still manage to survive through increased DNA repair processes or elevated apoptosis threshold. In addition, autophagy has a profound influence on platinum resistance. This review summarizes the critical participators of platinum resistance mechanisms mentioned above and highlights the most potential therapeutic targets or predicted markers. With a deeper understanding of the underlying resistance mechanisms, new solutions would be produced to extend the clinical application of platinum-based antitumor agents largely.

## Introduction

Ever since cisplatin, the first generation of platinum antitumor agents, was approved by the U.S. Food and Drug Administration for the treatment of testicular cancer, the development of platinum antitumor agents has explosively grown during the last forty years. The second-generation product carboplatin and the third-generation product oxaliplatin were approved worldwide in succession with enlarged spectrum or decreased toxicity (Wheate et al., 2010). Till today, cisplatin, carboplatin, and oxaliplatin are still extensively applied in the treatment of cancer.

**Figure 1:**
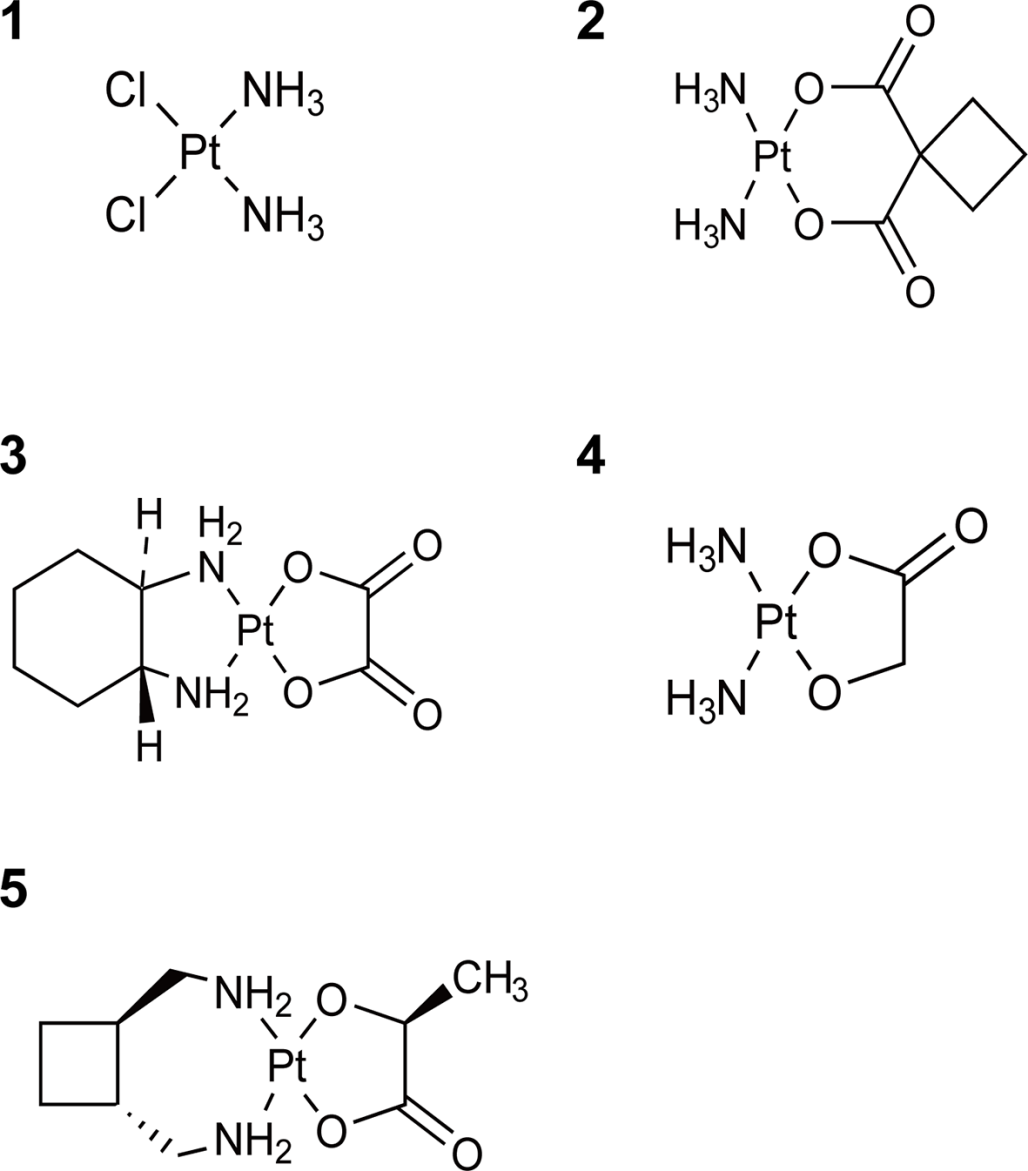
Chemical structures of platinum complexes. 1: Cisplatin; 2: Carboplatin; 3: Oxaliplatin; 4: Nedaplatin; 5: Lobaplatin.

Cisplatin (**Figure 1**) is a neutral, square planar coordination complex of platinum(II) coordinated to two chloride and two ammonia groups, where the chloride ligands are in the cis-geometry (Kartalou and Essigmann, 2001). Once inside the cell, cisplatin undergoes aquation to form [Pt(NH_3_)_2_Cl(OH_2_)]^+^ and [Pt(NH_3_)_2_(OH_2_)_2_]^2+^, therefore, it becomes more reactive to DNA (Jamieson and Lippard, 1999). It is approved for the treatment of advanced pancreatic cancer, breast cancer, non-small cell lung cancer (NSCLC), advanced bladder cancer et al. In the second-generation products, two chloride atoms are replaced by an oxygenated bidendate cyclobutane-dicarboxylate group to form carboplatin (cis-diammine-1,1′-cyclobutane dicarboxylate platinum II, **Figure 1**), which is easier to administrate and less toxic compared with cisplatin (McKeage, 1995). However, carboplatin has a similar anticancer spectrum with cisplatin, and the cross-resistance with cisplatin is observed in many cancer types. Oxaliplatin ({[oxalate(2-)-O, O′][1R,2R-cyclohexanediamine-N, N’] platinum-(II)}, **Figure 1**) belongs to the third generation of platinum-based antitumor agents, in which 1, 2-diaminocyclohexane (DACH) ligand substitutes for the amine groups of cisplatin. It was reported that oxaliplatin could produce fewer DNA adducts but caused higher cytotoxicity than cisplatin (Woynarowski et al., 2000), and it also shows stronger activity in colorectal and other gastrointestinal cancers, while cisplatin and carboplatin show no efficacy. Nedaplatin (cis-diammine (glycolato-O¹, O²)platinum, **Figure 1**), the second-generation platinum analogue, was first approved in Japan. It has approximately ten times as soluble in water as cisplatin and lower toxicity than cisplatin (Kuwahara et al., 2009; Zhong et al., 2018). Nedaplatin shows promising results in combination therapies (Li et al., 2017c; Peng et al., 2017; Takekuma et al., 2018). Lobaplatin (cis-[trans-1,2-cyclobutanebis(methylamine)][(S)-lactato-O^1^, O^2^]platinum(II), **Figure 1**), the third-generation platinum analogue, was approved in China for treatment of breast cancer, NSCLC, and chronic myelocytic leukemia (Wheate et al., 2010).

**Figure 2:**
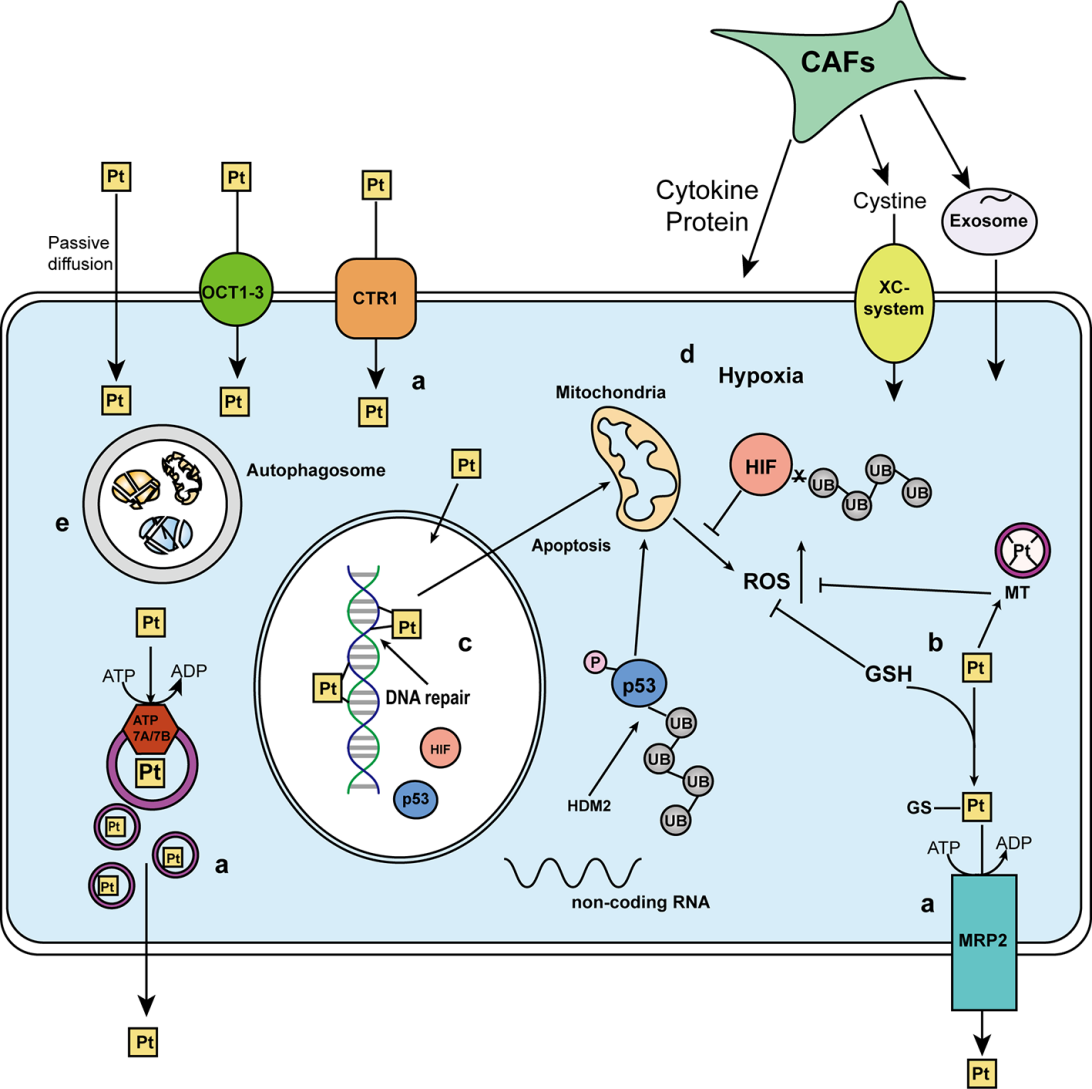
A schematic of the mechanisms affecting platinum response. The response toward platinum-based antitumor agents can result from **(a)** cellular drug accumulation. Besides passive diffusion, the uptake of platinum agents is mediated by multiple transporters. Organic cation transporters (OCT1-3) and CTR1 mediated the influx, while ATP7A/7B and MRP2 participate in the isolation and efflux of platinum agents or GS-platinum complex. **(b)** Detoxification system. Platinum agents can be deactivated by binding to detoxification components, glutathione (GSH) and metallothionein (MT). **(c)** DNA repair process. The platinum atom can covalently bound to the N7 positions of purine bases to form the platinum-DNA adducts and induce cytotoxicity, but the DNA repair process could repair the damaged DNA lesion. **(d)** Apoptosis. Once the DNA repair fails or is overwhelmed by too many DNA lesions, apoptosis will be triggered. Mitochondria will generate excessed reactive oxygen species (ROS) to kill the cells, which might be neutralized by GSH and MT. p53 and tumor microenvironment [including hypoxia-induced hypoxia-inducible factor (HIF) and cancer-associated fibroblasts (CAFs)] play key regulatory roles in apoptosis. **(e)** Autophagy, a self-digestion process, has two sides in affecting platinum response.

It’s common to see patients who respond well to platinum treatment become resistant to platinum-based chemotherapy in a short time. The resistance result from (i) reduced cellular drug accumulation, (ii) increased detoxification system, (iii) increased DNA repair process, (iv) decreased apoptosis, and (v) autophagy as summarized in **Figure 2**. This work reviews the updated platinum-resistance mechanisms and the key participators.

## Transporters Involved in Platinum Influx/Efflux

The accumulation of platinum antitumor agents inside the cells is the necessary assurance of cytotoxicity, so decreased influx or increased efflux is responsible for platinum resistance. For many years it has been assumed that platinum enters cells by passive diffusion and through gated channels (Gately and Howell, 1993). However, the role of active transport mediated by multiple transporters become prominent to platinum-uptake. Three urgent questions need explanations: (i) which transporters are responsible for platinum-uptake, (ii) how transporters change during drug-resistance development, and (iii) what can be done to reverse resistance by targeting transporters.

### Solute Carrier Superfamily of Membrane Transporters

The solute carrier superfamily (SLCs) contains more than 300 members and 65 subfamilies (Perland and Fredriksson, 2017) such as the organic anion transporting polypeptides, organic anion transporters, and organic cation transporters (OCTs). Normally SLCs are expressed in the whole body to sustain the cellular homeostasis by mediating the transportation of endogenous substances and exogenous substances. However, the expression or the distribution of SLCs may change under the disease condition or due to the drug–drug interaction effect, and it will have a significant effect on the cellular uptake of therapeutic drugs then leads to the unsatisfied results (Zhou et al., 2017).

Cisplatin is a substrate of hOCT1 (*SLC22A1*), hOCT2 (*SLC22A2*), and hMATE1 (*SLC47A1*), and oxaliplatin is a substrate of hOCT2, hOCT3 (*SLC22A3*), hMATE1 (*SLC47A1*), and hMATE2-K (*SLC47A2*). Carboplatin and Nedaplatin are not transported by the transporters mentioned above (Yonezawa et al., 2006). However, some contradictory results were reported probably because of the different selection of verification models, as summarized in **Table 1**. The down-regulation, mislocation, or inhibited transport activity of OCTs can all reduce the intracellular platinum concentration. Gao et al. reported that omeprazole could decrease the protein level of OCT2, thus lead to reduced cellular accumulation of cisplatin (Gao et al., 2019). Buss et al. (2018) employed a novel fluorescent oxaliplatin derivative to discovered that OCT1 involves in oxaliplatin uptake in the sensitive but not in the resistant cell line, which may be a consequence of the altered localization of the transporter in resistant cells rather than the lower expression. Recent studies reported two crucial regulatory mechanisms of OCTs: phosphotyrosine-mediated activity regulation and epigenetic regulation. Sprowl and colleagues revealed that the phosphorylation status of Y362, mediated by the Src family kinase Yes1, might be essential for OCT2 function; hence tyrosine kinase inhibitor like dasatinib can inhibit oxaliplatin uptake and mitigate oxaliplatin-induced acute sensory neuropathy (Sprowl et al., 2016). Our previous work revealed the link between the epigenetic changes of OCT2 and oxaliplatin resistance in renal cell carcinoma (RCC) (Liu et al., 2016; Zhu et al., 2019). We found that hypermethylated CpG islands of OCT2 disrupted the interaction between MYC and the E-Box motif, further it would prevent MYC recruiting MLL1 to catalyze H3K4me3 at the OCT2 promoter. As a consequence, the transcriptional repression of OCT2 diminished oxaliplatin accumulation and failed the oxaliplatin treatment in RCC. But decitabine, a demethylating reagent, can efficiently reverse the hypermethylation of OCT2. Therefore, the combination therapy of decitabine and oxaliplatin is a promising treatment option to reactivate RCC to oxaliplatin. The results indicated that OCT2 demethylation cracks open oxaliplatin resistance in RCC (Aguilar, 2016) and it highlights the huge potentiality of targeting transporters in clinical application.

**Table 1:** The influx/efflux transporters of cisplatin, carboplatin, oxaliplatin, nedaplatin, and lobaplatin.

	Solute carrier superfamily of membrane transporters							ATPases		ATP-binding cassette transporters			References
	SLC22 families			SLC47 families		SCL31A families							
	OCT1	OCT2	OCT3	MATE1	MATE2K	CTR1	CTR2	ATP 7A	ATP 7B	MRP1	MRP2	MRP4	(Holzer et al., 2006; Yonezawa et al., 2006); (Taniguchi et al., 1996; Borst et al., 2000; Samimi et al., 2004; Yokoo et al., 2007; Safaei et al., 2008; Blair et al., 2009; Beretta et al., 2010; Burger et al., 2010; Myint et al., 2015)
Cisplatin	±	₊	−	₊	−	₊	*	+	+	−	+	+	
Oxaliplatin	±	+	+	+	+	+	*	+	+	+	+	+	
Carboplatin	−	−	−	−	−	+	*	+	+	/	/	/	
Nedaplatin	−	−	−	−	−	/	/	/	/	/	/	/	
Lobaplatin	/	/	/	/	/	/	/	/	/	/	/	/	

+, substrate; −, not the substrate; ±, contradictory results; *, influenced accumulation but not proven transported substrates; /, not documented.

### Copper Transporter 1/2

Copper transporter 1 (CTR1) ubiquitously expresses in tissues and is required for high-affinity copper uptake. It is widely acknowledging that CTR1 transports cisplatin, oxaliplatin, and carboplatin (Holzer et al., 2006). Deletion of the yeast CTR1 gene reduces the intracellular accumulation of cisplatin and leads to cisplatin-resistance (Ishida et al., 2002), while forced overexpression of hCTR1 sensitizes small cell lung cancer cells to cisplatin, carboplatin, and oxaliplatin (Song et al., 2004). Therefore, hCTR1 has the potentiality to become a hopeful biomarker for platinum-based chemotherapy. It can be down-regulated by intracellular copper (Howell et al., 2010) or cisplatin/oxaliplatin treatment which triggers a rapid loss of hCTR1 *via* macropinocytosis and proteasomal degradation in ovarian carcinoma cells (Holzer and Howell, 2006) and hepatocellular carcinoma cells (Li et al., 2016b). Ishida et al. shown that copper chelator tetrathiomolybdate could increase the uptake of cisplatin into tumor cells specifically (Ishida et al., 2010), and so did Fu and coworkers. They reported a combined therapy of carboplatin and trientine, a copper-lowering agent, partly reversed resistance to platinum therapy on five patients with platinum-resistant high-grade epithelial ovarian cancer (Fu et al., 2012). The proteasome inhibitors bortezomib (Al-Eisawi et al., 2013) and natural compound β-elemene were also discovered that could block CTR1 from degradation (Li et al., 2016b). However, a lot of conflicting conclusions of whether regulating CTR1 levels affects sensitivity to platinum-based drugs have emerged. Kristin et al. (Bompiani et al., 2016) knocked out the CTR1, CTR2, ATOX1, and CCS using CRISPR-Cas9 genome editing; and the results indicated that the loss of CTR1, CTR2, ATOX1, or CCS had little impact on cisplatin sensitivity in both human HEK-293T and ovarian carcinoma OVCAR8 cells. Another research found that overexpression of CTR1 (Akerfeldt et al., 2017) failed to increase platinum accumulation and had no effect on the sensitivity of cisplatin in DLD-1 cells. The clinical relevance of hCTR1 and platinum-based chemotherapy has been questioned as well (Kim et al., 2014).

CTR2 is a low-affinity transporter of copper that shares 41% amino acid homology and the similar essential domains for copper transport with CTR1 except for the extended N-terminal domain (Gupta and Lutsenko, 2009). It locates at late endosomes and lysosomes, although it had also been found on the plasma membrane (van den Berghe et al., 2007). The mRNA and protein levels of hCTR2 have significant correlations with the sensitivity of cisplatin (Blair et al., 2009). Knocking down CTR2 in some cells increases the cellular accumulation of cisplatin, yet overexpressing the CTR2 reduces the sensitivity to cisplatin (Huang et al., 2014). Blair et al. indicated that CTR2 regulated the accumulation of cisplatin through an effect on macropinocytosis, not by changing drug efflux or microsomal storage (Blair et al., 2011). Moreover, CTR2 can interact with CTR1 *via* stimulating CTR1 ectodomain cleavage resulting in less accumulation of cisplatin in cells (Ohrvik et al., 2016).

### ATP7A and ATP7B

ATP7A/7B, which belongs to P-type ATPases, is responsible for copper homeostasis (Gupta and Lutsenko, 2009). After getting into the cells, platinum may bind to the CXXC motifs of ATP7A/B (Safaei et al., 2012), then the complex translocates into a vesicle in an ATP-dependent manner with the association of copper chaperone Atox1 (Boal and Rosenzweig, 2009). ATP7A/B resides in the trans-Golgi network under normal conditions (Hall et al., 2008; Kalayda et al., 2008), but in platinum-resistance cells, it distributes in more peripherally located vesicles in the cytosol. The altered localization may be caused by reduced lysosomal compartment, and it contributes to platinum-sequestration (Kalayda et al., 2008). ATP7A acts as an insulator, keeping cisplatin away from nuclear in resistance cells. Chisholm and coworkers observed that the cellular platinum intensity is low and is excluded from the nucleus (Chisholm et al., 2016) when it shows high expression. ATP7B is also regarded as a contributor to platinum resistance and may serve as a prognostic factor. Patients with the lowest mRNA expression levels of ATP7B presented a significantly longer time to progression and had the optimal curative effects from oxaliplatin/5FU treatment in colorectal cancer (Martinez-Balibrea et al., 2009). Given the above, it seems that downregulating ATP7A/B could be an effective way to overcome the resistance in tumor cells. By direct binding to 3′ untranslated region (3′ UTR) of the ATP7A/B mRNA, some microRNAs (miRNA, miR) regulates the cell response to cisplatin, carboplatin, and oxaliplatin such as miR-495, miR-139, and miR-133a (Song et al., 2014; Wang et al., 2016c; Xiao et al., 2018). The copper chelator ammonium tetrathiomolybdate ([(NH_4_)_2_MoS_4_], TM) can also restore the sensitivity to cisplatin by inducing dimerization of the metal-binding domain of ATP7B (Fang et al., 2019).

### Multidrug Resistance Protein Subfamily

Multidrug resistance protein (MRP, ABCC) subfamily, which has seven subfamilies named A to G, belongs to the ATP-binding cassette transporter superfamily and functions as an ATP-dependent unidirectional efflux pump for anionic amphiphilic compounds (Jedlitschky et al., 1994; Yaneff et al., 2019). MRPs mediated the efflux of endogenous molecules, physiological substrate, and drugs (reviewed in Keppler, 2011). MRP2, not MRP1 or MDR1, has long been recognized as the efflux transporter of the platinum-GSH conjugate (Taniguchi et al., 1996; Borst et al., 2000). High-level expression of MRP2 is associated with intrinsic cisplatin resistance and clinical outcome in small cell lung carcinoma (Ushijima et al., 2007), ovarian cancer (Surowiak et al., 2006), and esophageal squamous cell carcinoma (Yamasaki et al., 2011). MRP4 has also been identified as a platinum resistance-associated candidate. The overexpression of MRP4 is related to cisplatin resistance (Zhang et al., 2010), and knocking down MRP4 instead of MRP1 increases the accumulation of oxaliplatin and cisplatin (Beretta et al., 2010; Zhang et al., 2010).

## Detoxification System

### Glutathione

Glutathione (L-γ-glutamyl-l-cysteinyl-glycine, GSH) plays a vital role in the cellular redox state *via* scavenging free radicals, defending cells against xenobiotics and maintaining the sulfhydryl groups of many proteins (Brozovic et al., 2010). The active SH-group of GSH has a high affinity to platinum, thus makes GSH become an easy, non-DNA-related target (Zimmermann and Burda, 2010). The GS-platinum complex catalyzed by glutathione-S-transferase can reach to about 60% of the intracellular platinum content after 12-h incubation in leukemia cells (Ishikawa and Ali-Osman, 1993), and the elevated expression of GSH and glutathione-S-transferase are often seen in the resistant-cells (Masters et al., 1996; Byun et al., 2005). Preventing the formation of the GS-platinum complex may reverse the platinum resistance efficiently. One choice is to use competitive inhibitors of GSH such as [Cu(phen)(H_2_O)_2_(ClO_4_)_2_] (C10) (Cadoni et al., 2017), another is to interfere with the synthesis of GSH. Two enzymes mediate the production of GSH, γ-glutamylcysteine ligase and GSH synthetase. In the first step, γ-glutamylcysteine ligase catalyze glutamate and cysteine to form γ-glutamylcysteine; then, in the second step, GSH synthetase catalyzes γ-glutamylcysteine and glycine to form GSH (Aoyama and Nakaki, 2013). GSH-depleting agents buthionine sulfoximine is a useful agent for inhibition of γ-glutamylcysteine ligase, and it reduced the resistance to cisplatin in malignant glioma (Rocha et al., 2015). However, non-specific GSH depletion can cause irreversible damage in most normal tissues. Hence selective tumor GSH depletion appears to be a better choice but remains as a superb challenge (reviewed in Estrela et al., 2006). Cystine is normally used as the precursor for GSH synthesis due to the chemical instability of cysteine, and the intracellular cysteine/cystine level is maintained by the xc^−^ system which mediates the uptake of cystine; so finding ways to inhibit xc^−^ system can block the synthesis of GSH. Sulfasalazine (Ma et al., 2015b) and salubrinal (Wang et al., 2018) are both reported as powerful blockers. Epigenetic interference also involves in the regulation of the GSH synthesis: miRNA-27a negatively regulates the xc^-^ (Drayton et al., 2014); G9a, a transcriptional corepressor that catalyzes histone 3 lysine 9 dimethylation, transcriptional activates the glutamate-cysteine ligase catalytic subunit, and lead to the elevation of GSH level in head and neck squamous cell carcinoma (Liu et al., 2017a); Linc RNA (Lnc RNA, Lnc) H19 participates in the production and regeneration of GSH in high-grade serous ovarian cancer (Zheng et al., 2016).

GSH redox cycle also regulates platinum resistance. GSH can be oxidized into GSSG by GSH peroxidase using H_2_O_2_ as a substrate, whereas GSSG can be reduced back to GSH by GSSG reductase using NADPH as a cofactor (Chen and Kuo, 2010). Higher expression of glutathione reductase accompanied by lower levels of endogenous reactive oxygen species (ROS) contributes to the cisplatin-resistance (Zhu et al., 2018).

### Metallothionein

Platinum antitumor drugs are also inactivated by chelating with metallothionein (MT) proteins. MTs are low-molecular-weight metal-binding proteins containing one-third cysteine residues, which make MTs become easy targets for platinum to chelate (Kimura and Kambe, 2016). Platinum agents may also bind to metal transcription inhibitor and release the metal transcription factor-1 to trigger the biosynthesis of MTs (Krizkova et al., 2010). It has been found that MTs level is increased not only in the tumor tissue but also in the serum of cancer patients (Tariba et al., 2015); RNA interference can inhibit the overexpression of MTs efficiently and reverse platinum resistance (Lee et al., 2015). Expressing level of the MTs has been considered as an important biological factor of platinum-based chemotherapy (Yamamoto et al., 1999), but rather than global MTs classes, Habel and colleagues suggested that MT isoforms are more valuable prognosis predictors markers (Habel et al., 2013). Pekarik et al. noted that MT-1 and MT-2 have redundant binding sites for many miRNAs, including miR23 and miR224 (Pekarik et al., 2013). It indicated that miRNAs may involve in the regulation of MTs, and could be a notable pointcut.

## DNA Repair

The platinum atom can covalently bound to the N7 positions of purine bases to form the platinum–DNA adducts. Cisplatin can form mono adducts, intrastrand, or interstrand cross-links, but the 1,2-intrastrand crosslinks account for over 90% (reviewed in Jamieson and Lippard, 1999; Chaney et al., 2005). It is well acknowledged that forming DNA adducts is the determinant step for cytotoxicity of platinum-based antitumor agents because the platinum–DNA complexes influence the structure of DNA double helix (Kartalou and Essigmann, 2001) and nucleosomes (Danford et al., 2005). As a result, it causes replication and transcription inhibition and DNA double-strand breaks (DSBs), followed by the initiation of DNA repair. Once the DNA repair fails or is overwhelmed by too many DNA lesions, cell death will be triggered (reviewed in Jung and Lippard, 2007). The increased DNA repair process is considered as the most significant characteristic in platinum-resistance cells (Dijt et al., 1988; Wynne et al., 2007), with the exception of DNA mismatch repair whose deficiency gives rise to cisplatin/carboplatin resistance (Sawant et al., 2015), but has less influence to oxaliplatin (Goodspeed et al., 2019). Oxaliplatin has more potent cytotoxicity than cisplatin for inducing early secondary DSBs and massive apoptosis (Faivre et al., 2003). However, Bruno et al. reported that oxaliplatin induces ribosome biogenesis stress rather than DNA damage to kill cells (Bruno et al., 2017). Most intrastrand crosslinks are removed by nucleotide excision repair (NER) system *via* excising damaged nucleotides and synthesizing DNA to reconstitute genetic integrity, while other lesions are repaired by complex-combined mechanisms (Roos and Kaina, 2013). Only two key components of the DNA repair system will be discussed in this review.

### Excision Repair Cross-Complementing

All excision repair cross-complementing (ERCC) members have their unique roles in DNA repair progress, and their expression level or SNP has a significant impact on platinum resistance. In a prospective study, Sullivan et al. analyzed the SNPs in eight DNA-repair related genes and found that after the treatment of platinum-based chemotherapy, the response of patients with stages III significantly associated with SNPs in ERCC1 and ERCC3 genes, while the response of patients with stage IV associated with a genetic variant in the ERCC4 gene (Sullivan et al., 2014). Many researchers highlight the relevance between ERCC1 and platinum-treatment response (Li et al., 2000; Ozcan et al., 2013). Liu et al. found that under DNA damage condition, p53 recruited CITED2/P300 along with chromatin relaxation H3K9Ac or H3K14Ac to bind with the ERCC1 promotor, and activated the DNA repair process (Liu et al., 2015b). However, Spada et al. reported that ERCC1 had no significant correlation with the clinical outcome with oxaliplatin-based chemotherapy in advanced neuroendocrine tumors (Spada et al., 2016). ERCC4 (XPF), a necessary component in NER, interstrand cross-links repair, homologous recombination repair (HRR) but not in non-homologous end-joining (Lehmann et al., 2017), contributes to the intrinsic resistance of cisplatin, and its expression level is tissue-specific (Zhang et al., 2016a). ERCC1 and XPF can form the human ERCC1–XPF complex which works as a nuclease in NER and the late stage of HRR (Gillet and Scharer, 2006; Al-Minawi et al., 2009). Arora et al. reported that knocking down ERCC1–XPF resulted in reduced intrastrand repair and interstrand crosslinks repair, which makes about fourfold to sixfold changes in IC_50_ value of cisplatin in NSCLC cells (Arora et al., 2010). Based on the above results, it is reasonable to believe that inhibiting ERCC1/XPF is a potential way to sensitize tumor cells to platinum-based chemotherapy, and many inhibitors are discovered: green tea polyphenol epigallocatechin-3-gallate, catechols, 3-hydroxypyridones, N-hydroxyimides, and hydroxypyrimidones (Chapman et al., 2015; Wang et al., 2015), but their clinical application still needs further investigation. Another nuclease function as ERCC1–XPF in NER is ERCC5 (XPG), which also relates to platinum sensitivity. Graf et al. reported that XPF and XPG knockdown increased the platinum-induced cytotoxicity in osteosarcoma cells (Graf et al., 2011). Interestingly, platinum-based chemotherapy is influenced by the 5′ noncoding mRNA element of ERCC5. Somers’s group discovered a common polymorphic variant rs751402 in the ERCC5 5′ untranslated region, which generated an upstream ORF, and the carriers with early childhood ependymoma were markedly resistant to platinum-based agents (Somers et al., 2015).

### Breast Cancer Susceptibility Gene

Breast Cancer Susceptibility Genes (BRCAs) involve in the repair of DSB and DNA cross-linking damage induced by DNA-damaging agents through the HR pathway (reviewed in Foulkes and Shuen, 2013); BRCA1/2-deficient carcinomas have impaired HRR and become more sensitive to platinum agents (Dann et al., 2012; Muggia and Safra, 2014). The alterations or mutations of BRCA1 and BRCA2 lead to the impaired recognition of DNA damage, and therefore depending on the patients’ BRCA status to add platinum agents into neoadjuvant therapy before surgery could receive an excellent response (Golan et al., 2014; Soyano et al., 2018). However, in BRCA mutant cancers, platinum resistance still exists. To explain that phenomenon, Guillemette et al. conducted a genome-wide shRNA screen and found that loss of the nucleosome remodeling factor CHD4 might cause cisplatin resistance through a trans-lesion synthesis dependent manner rather than HRR in BRCA2 mutant cancer cells(Guillemette et al., 2015). In addition, a secondary mutation in BRCA1/2 may occur after patients receiving platinum-based chemotherapy treatment, and it restores the function of BRCA1/2, ending with platinum resistance (Norquist et al., 2011). Researchers recently found two regulators: Wwox and deubiquitination enzyme USP13. Wwox can interact with BRCA1 and tip the DSBs repair pathway choice, and its deficiency permits significantly increased resistance to cisplatin, so it could be an efficient predictor of response to cisplatin (Schrock et al., 2017). The knockdown of USP13 dramatically diminishes cisplatin-induced RAP80-BRCA1 foci formation, for its deficiency decreases the bind between RAP80 and K63-linked ubiquitin chain and fail the BRCA1 recruitment (Li et al., 2017b).

Other than ERCC and BRCA, many key components like FANCD2 (Martens-de Kemp et al., 2017), PCNA (Somasagara et al., 2017), XRCC1 (Xu et al., 2014), and RAD51 (Pan et al., 2013b; Xiao et al., 2017), also play different roles in multiple DNA repair pathway. A better understanding of the DNA repair regulation network will provide a more accurate guide to personalized platinum-based chemotherapy.

## Apoptosis

After binding to the DNA, the core downstream events platinum agents triggered is apoptosis, which is also called programmed cell death. Two main apoptosis pathways have been well proposed: the extrinsic and the intrinsic pathways. The extrinsic pathway is activated after the tumor necrosis factor family binding to the cell surface receptors of the tumor necrosis factor receptor superfamily and leads to the self-activation of initiator caspase-8. The intrinsic pathway is switched by an imbalance between proapoptotic (i.e., BAX, BAK) and antiapoptotic proteins (i.e., BCL-2, BCL-XL, BCL-w). Upon activation, proapoptotic signaling leads to mitochondrial outer membrane permeabilization, and then cytochrome c will be released, initiating a series caspase cascade (Kim et al., 2002; Bai and Wang, 2014). Platinum-resistant tumor cells usually have a higher threshold for apoptosis induction, mostly due to the overexpression of anti-apoptotic proteins or the defect in mitochondrial signaling. Many factors contribute to these unwanted phenomenons, such as pro-survival signal pathways (Sun et al., 2016; Bao et al., 2017) [i.e., MAPK/ERK (Kong et al., 2015), PI3K/AKT pathway, NF-κB (Miow et al., 2015)], and tumor microenvironment (TME) and epigenetic regulation (Benard et al., 2014; Ramadoss et al., 2017). Researchers begin focusing on the critical role of non-coding RNA in diverse cellular processes; studies demonstrated that non-coding RNA broadly participated in proapoptotic/antiapoptotic proteins regulation and could work as therapeutic targets or predict markers (Chen et al., 2015; Zarogoulidis et al., 2015). We summarized recently found apoptosis-related non-coding RNA with details listed in **Table 2**. Among all the contributors, we specifically concentrate on the role of p53 and TME in platinum resistance.

**Table 2:** The LncRNAs and miRNAs regulate platinum resistance through targeting apoptosis and autophagy in various cancer.

			Target	Platinum agents	Cancer type	References
Apoptosis	LncRNA	LncRNA UCA1	miR-184/SF1	Cisplatin	Oral squamous cell carcinoma	(Fang et al., 2017)
			CREB-miR-196A-5P		Bladder cancer	(Pan et al., 2016)
		LncRNA AC023115.3	miR-26a-GSK3β		Malignant glioma	(Ma et al., 2017)
		LncRNA XIST	let-7i/BAG-1		Lung adenocarcinoma	(Sun et al., 2017b)
		LINC00473	C/EBPβ IL24		Osteosarcoma	(Zhang et al., 2017)
		HOMEOBOX A11	miR‐454‐3p/Stat3		Lung adenocarcinoma	(Zhao et al., 2018a)
		HOTAIR	PI3K/Akt Wnt/β-catenin miR-34a		Gastric cancer	(Cheng et al., 2018)
		Lnc PVT1	HIF1α			(Zhang et al., 2015)
	miRNA	miR−34a	MAGE-A/p53	Carboplatin	Retinoblastoma	(Yang et al., 2019)
		miR-205 miR-218	Mcl-1 Survivin		Lung cancer	(Zarogoulidis et al., 2015)
		miR-634	Ras-MAPK		Ovarian cancer	(van Jaarsveld et al., 2015)
		miR-139-5p	MAPK	Cisplatin	Ovarian cancer	(Chen et al., 2018)
		miR-5100	Rab6		Lung cancer	(Yang et al., 2018)
		miR-873	Bcl-2		Gliomas	(Chen et al., 2015)
		miR-199a-3p	ZEB1		Melanoma	(Liang et al., 2018)
		miR-195	Prohibitin 1			(Cirilo et al., 2017)
		miR-125a-5p	STAT3		Esophageal carcinoma	(Zhao et al., 2018b)
		miR-374a	PDCD4		Nasopharyngeal carcinoma	(Zhen et al., 2017)
		miR-146a	Cyclin J		Non-small cell lung cancer	(Shi et al., 2017)
		miR-148b	DNMT1			(Sui et al., 2015)
		miR-200c	AKT2		Osteosarcoma	(Liu et al., 2017d)
		miR-378	Clusterin		Lung adenocarcinoma	(Chen et al., 2016)
		miR-216b	PARP1		Ovarian cancer	(Liu et al., 2017c)
		miR-148a	Rab14		Renal cell carcinoma	(Kim et al., 2017a)
		miR-126	SERPINE1 SLC7A5 mTOR/HIF			(Liu et al., 2017b)
		miR-17-92	AKT		Prostate cancer	(Zhou et al., 2016b)
		miR-99a miR-491	CAPNS1		Gastric cancer	(Zhang et al., 2016b)
Autophagy	LncRNA	BLACAT1	miR-17/ATG7	Cisplatin	Non−small cell lung cancer	(Huang et al., 2019)
		MALAT1	microRNA−30b/autophagy−related gene 5		Gastric cancer	(Xi et al., 2019)
	miRNA	miR-634	APIP/XIAP/BIRC5/OPA1 NRF2	Cisplatin	Esophageal squamo-us cell carcinoma	(Fujiwara et al., 2015)
		miR-148A-3p	RAB12 mTORC1		Gastric cancer	(Li et al., 2017a)
		miR-874	ATG16L1			(Huang et al., 2018)
		miR-let-7f-1	HMGB1		Medulloblastoma	(Pannuru et al., 2014)
		miR-181	PTEN/PI3K/AKT/mTOR		Non-small cell lung cancer	(Liu et al., 2018)
		miR-409-3p	Beclin-1	Oxaliplatin	Colon cancer	(Tan et al., 2016)
		miR-218	YEATS4		Colorectal cancer	(Fu et al., 2016)
		miR-34a	TGF-β/Smad4 pathway			(Sun et al., 2017a)

LncRNA, long non-coding RNA; miRNA, microRNA.

### p53

The tumor suppressor p53 functions mainly as a transcriptional activator of many cellular programs, including checkpoint activation, DNA repair, and apoptosis, and therefore the status of p53 is crucial for the cytotoxicity of platinum agents (Gadhikar et al., 2013). Downstream genes of p53 related with apoptosis include FAS, BBC3, BAX, and BIRC5 (Riley et al., 2008). Besides the nuclear function, p53 located in cytoplasmic also promotes apoptosis through directly targeting mitochondria (reviewed in Marchenko and Moll, 2007). However, it is common to see the loss or mutation of p53 in the patients (Ahmed et al., 2010; Stransky et al., 2011), and the disfunction of p53 will cause the failure of checkpoint response, cell cycle arrest, programmed cell death (apoptosis), and permanent cell cycle arrest (senescence) (Martinez-Rivera and Siddik, 2012). Eventually, it leads to a poor outcome of platinum-based chemotherapy. A thought-provoking work reported a nonsynonymous single-nucleotide polymorphism Pro47Ser (rs1800371) in African descent; the carriers are more tumor-prone and show a significant defect in cisplatin-induced cell death due to the impaired trans-activation ability (Jennis et al., 2016). For this reason, more precision medicine approaches should be considered for different mutation carriers (Basu et al., 2016).

Antirepression, stabilization, DNA binding, and transcriptional activation are four key steps in p53’s activation along with an exquisite regulation network including phosphorylation, ubiquitination, methylation, sumoylation, neddylation, and acetylation (reviewed in Kruse and Gu, 2009). Ubiquitinate-dependent degradation of p53 is mediated by human double minute 2 (HDM2, MDM2); therefore, interference with the HDM2/p53 pathway is an optional strategy to rescue p53 from degradation and reactivate it. Two p53-MDM2 blockers, RITA (Issaeva et al., 2004) and nutlin (Vassilev et al., 2004), were discovered in 2004. Furthermore, recently Wanzel and coworkers used CRISPR-Cas9 based target validation to reveal the actual mechanisms of two p53-MDM2 blockers (Wanzel et al., 2016). Nutlin was designed to blocks the p53-binding pocket of Mdm2 and RITA was to bind the Mdm2-interacting N terminus of p53, but on the contrary, results showed that the activity of nutlin strictly depends on functional p53 while the sensitivity of RITA correlates with induction of DNA damage but not with p53 (Wanzel et al., 2016). The phosphorylation status is also vital to functional p53. DACH-diacetato-dichloro-Pt(IV) (DAP) and oxaliplatin circumvent cisplatin resistance for phosphorylating p53 at Ser20 in resistant cells while cisplatin cannot (Xie et al., 2017), and in cisplatin-resistant MCF-7 cells, resveratrol induces serine 20 phosphorylation to activate p53 target genes such as PUMA and BAX, restoring apoptosis (Hernandez-Valencia et al., 2018). All these results demonstrate that phosphorylation at Ser 20 site might be a critical event for p53 activation.

### TME

The TME is a complex ecosystem and an active regulator in the progression of cancer development. The interaction between TME and cancer cells is mutual and dynamical. Tumor epithelial cells can gradually change the nature of the microenvironment, such as hypoxia, and conversely, the altered TME will regulate tumor development (Belli et al., 2018). TME consists of nonmalignant cells of the tumor such as cancer-associated fibroblasts (CAFs), endothelial cells and pericytes composing tumor vasculature, immune and inflammatory cells, bone marrow-derived cells, and the extracellular matrix (Belli et al., 2018). Some have been proven participating in the formation of drug resistance.

#### CAFs

The CAFs, which held the main proportion of tumor stroma, are perpetually activated, and neither revert to a normal phenotype nor undergoes apoptosis and elimination (Li et al., 2007). Emerging evidence demonstrates that non-malignant CAFs can contribute to tumor proliferation, metastasis, and chemoresistance. CAFs secrete specific cytokines, proteins, or exosomal miRNA to activate certain anti-apoptosis related signaling pathways like PI3K/Akt, ANXA3/JNK, and IL-11/IL-11R/STAT3, and consequently offer tumor cells the ability of resistance (Tao et al., 2016; Zhou et al., 2016a; Zheng et al., 2017b; Qin et al., 2019; Wang et al., 2019b). CAFs can also cause the aberrant remodeling of extracellular material and physical properties of the tumor altered, or release cysteine and GSH to limit the intracellular platinum concentration (Akkari and Joyce, 2016; Wang et al., 2016b). It is worth mention that most of these researches focus on the resistance change of tumor cells after platinum treatment. However, the innate chemoresistant of CAFs is poorly understood. Qin X et al. recently reported that HNC-derived CAFs were innately resistant to cisplatin, maybe due to the high expression of ERCC1 and ERCC4 (Qin et al., 2019).

#### Hypoxia

The imbalance between the rapid growth of tumor cells and inadequate supply from blood vessels alters the TME, and one of the significant characteristics is hypoxia (Bertout et al., 2008), which is strongly associated with a poor prognosis in different cancer types, such as hepatocellular carcinoma (Dai et al., 2009), ovarian cancer (Osada et al., 2007), NSCLC (Hung et al., 2009), etc. Hypoxia triggers the regulation of hypoxia-inducible factor (HIF) family at the transcription level and protein level (Pawlus and Hu, 2013). HIFs are heterodimeric transcription factors composed of two parts: an oxygen-labile alpha subunit (HIF1α, HIF2α, or HIF3α) and a stable beta subunit (HIF1β). HIF1α and HIF2α are widely expressed in various tissues and have been well understood, yet HIF3α is less known due to its high specificity of tissue distribution and the existence of multiple variants (Duan, 2016). So only HIF1α and HIF2α will be discussed in this review. Under normoxic conditions, two specific proline residues in HIF-1α are hydroxylated by HIF prolyl hydroxylase enzymes with the cofactor oxygen, iron, ascorbate, and 2-oxoglutarate. Then, the hydroxylated HIF1α protein is bound with the von Hippel–Lindau protein, and it will recruit E3 ubiquitin ligases and put HIF1α through polyubiquitination and degradation by the 26S proteasome. Whereas under hypoxic conditions, impaired hydroxylation process stabilizes the HIF1α protein, furthermore HIF1α will translocate into nuclear and dimerize with HIF1β to transactivate the target genes which relate with glucose metabolism, angiogenesis, cell proliferation, invasion and metastasis (reviewed in Pawlus and Hu, 2013; Soni and Padwad, 2017). HIF1α has a strong association with apoptosis; it regulates apoptosis-related genes (i.e., Bcl-2, Bax, caspase 3, caspase 8) (Hernandez-Luna et al., 2013; Zhao et al., 2016), and survival signaling pathways such as NF-κB (Rohwer et al., 2010). Zheng et al. recently found that HIF-1α could activate the PI3K/AKT by up-regulating Mxd1 expression to suppress the PTEN expression (Zheng et al., 2017a). Besides, HIF accumulation facilitates the secretion of several growth factors and further activates mTOR to form a positive feedback circuit. Deactivating these signaling pathways *via* reducing HIFs could be a promising approach.

Highly proliferative cancer cells switch glucose flux from oxidative phosphorylation to anaerobic glycolysis, which converts pyruvate into lactate even in the presence of oxygen (the Warburg effect). This shift will quench cytosolic ROS (Hervouet et al., 2007). However, recent evidence shows that in some cytotoxic antineoplastic drug-resistance tumor cells, aerobic glycolysis can shift back to oxidative phosphorylation (Xu et al., 2018). HIFs act as inducers to aerobic glycolysis and a suppressor to mitochondrial function; it can regulate many main enzymes in glycolysis and tricarboxylic acid cycle like GLUT1, LDH-A, PDH, and PDK1 (Nagao et al., 2019). Downregulating HIF-1α to redirect the aerobic glycolysis towards oxidative phosphorylation resulting in overexpressing ROS can resensitize the cisplatin-resistance ovarian cancer cells (Ai et al., 2016). ROS act as a double-bladed sword in the platinum treatment. On the one hand, platinum agents-induced cytotoxicity is strongly linked to excessed ROS generation (Brozovic et al., 2013; Choi et al., 2015; Jiang et al., 2015; Li et al., 2016c). Unlike cisplatin, oxaliplatin prefers to stimulate O_2_^–^ production rather than H_2_O_2_ (Chen et al., 2017). Some essential components were newly discovered. Wang et al. reported that Scribble promotes cisplatin-induced apoptosis *via* protecting Nox2/ROS (Wang et al., 2019a). Inositol 1, 3, 4, 5-tetrakisphosphate, the product of inositol-trisphosphate 3-kinase B, inhibits NADPH oxidase 4, thereby inhibiting the generation of cisplatin-induced ROS (Pan et al., 2019). The imbalance between ROS production and elimination is often seen in resistance cells. Besides GSH and MTs, the elevated oxidative branch of the pentose phosphate pathway is another strong defensor against ROS (Xu et al., 2018). The key, it seems, is whether the ROS level can reach the threshold to trigger cell death. Stimulating the production or blocking the neutralization of ROS might be a promising way to reverse platinum resistance. On the other hand, the increased ROS level in cancer cells might accelerates the development of tumor aggressiveness and drug resistance (Kumar et al., 2008). Increased ROS can activate cellular survival pathways including c-Myc–miR-137–EZH2 pathway (Sun et al., 2019), IL-11–JAK2–STAT5 pathway (Zhou et al., 2018), ATR–Chk1 pathway (Meng et al., 2018) and AXL (Oien et al., 2017) et al. ROS may also mediate metabolism reprogramming (Cruz-Bermudez et al., 2019).

Classical strategies include direct/indirect HIF targeting and interference in upstream/downstream HIF regulators/signaling pathways. Many prodrugs, specific HIF inhibitors, and non-selective inhibitors are developed to inhibit HIFs with many different mechanisms including inhibition of HIF dimerization, mRNA or protein expression, transcriptional activity, and DNA binding capacity (reviewed in Wigerup et al., 2016). Recently found Trx-1 and KLF5 (Li et al., 2016a) are upstream regulators of HIF 1α. Trx-1 increase the expression and the binding capacity of HIF1α through enhanced interaction with Ref-1 and inhibition of Trx-1/Ref-1 axis can strengthen the oxidative phosphorylation, and reverse the resistance to cisplatin (Zhao et al., 2015). Gong et al. reported KLF5 knockdown suppressed hypoxia-induced cisplatin resistance by inhibiting HIF1α-dependent glycolysis through inactivation of the PI3K/Akt/mTOR pathway (Gong et al., 2018). Non-coding RNAs can also increase the expression of HIF1α, resulting in platinum resistance (Zhang et al., 2015; Liu et al., 2017b).

Besides HIF1α, HIF2α also associate with platinum response. GBM cells show an increase in cisplatin resistance after exposure to hypoxia, and downregulating HIF2α, not HIF1α, significantly sensitizes U251 and U87 cells (GBM cell lines) to cisplatin (Ahmed et al., 2018). Knocking down HIF2a significantly increases the sensitivity to cisplatin in A549 cells (Gao et al., 2018).

## Autophagy

Autophagy, a “self-digestion” process, occurs in all eukaryotes. It is essential for nutrient regulation and intracellular quality control and homeostasis (Mizushima and Klionsky, 2007). Also, autophagy functions as a self-defense strategy by recycling macromolecules as an alternative energy source. However, if autophagy continuously or excessively proceeds, cell death will be triggered. There are generally three major types of autophagy, macroautophagy, microautophagy, and chaperone-mediated autophagy. In this review, only macroautophagy (referred to as autophagy) will be discussed. Macroautophagy seems to have a conflicting role in tumorigenesis and progression. It can act as a prodeath or prosurvival role at different cancer stages (reviewed in Dalby et al., 2010). After platinum treatment, the increased drug-induced autophagy is observed in platinum-resistant cells, along with the increased basal autophagy (Yu et al., 2014; Liu et al., 2015a; Wu et al., 2017; Gao and Wang, 2019; Wang et al., 2019c). Inhibition of autophagy *via* autophagy inhibitor or interference in regulatory elements or noncoding RNAs has been proven can reduce the platinum resistance. Using 3-methyladenine or chloroquine to inhibit early/later-stage autophagy can both enhance platinum-mediated cytotoxicity (Fukuda et al., 2015; Ma et al., 2015a). Autophagy is highly controlled by regulatory elements including PI3K–Akt–mTOR pathway, Beclin1, Bcl-2, Ras, p53, and noncoding RNAs [long non-coding RNAs (LncRNs) and miRNAs] (reviewed in Kumar et al., 2015). Recently, new regulators dual-specificity protein phosphatase 1 (DUSP1) (Wang et al., 2016a), heparinase (Shteingauz et al., 2015), and HMGB1 (Liu et al., 2015a), GFRA1 (Kim et al., 2017b) are reported. Targeting these regulatory elements can restore the efficiency of platinum agents. LncRNAs and miRNAs affect the platinum resistance by targeting key components like ATG7 or signaling pathways like PI3K/AKT/mTOR in autophagy. We summarized recently found autophagy-related non-coding RNA with details listed in **Table 2**.

Another big issue caused by autophagy is related to cancer stem cells (CSC). CSCs are a subpopulation of cancer cells within the tumor that have the ability to self-renew and to differentiate. CSCs have long been considered as the main culprit for drug resistance and relapse (MacDonagh et al., 2016; Steinbichler et al., 2018). Growing evidence indicated that autophagy protects CSCs for participating in the regulation of stem cell differentiation, somatic reprogramming and self-renewal capacity (Pan et al., 2013a). Yang et al. reported that oxaliplatin-induced autophagy enriches the population of colorectal CD44^+^ CSCs and maintain the stemness of colorectal CSCs, this protection eventually causes chemoresistance in colorectal CSCs (Yang et al., 2015), and so did Nail’s group. They found that autophagy regulates the expression of stemness surface marker CD44, drug resistance marker ABCB1 and invasion mediator ADAM17 to help maintain stemness and chemoresistance, but the mechanism remains unclear (Naik et al., 2018). Targeting autophagy to diminish the CSCs subpopulation might be a new direction to overcome platinum resistance.

In most aforementioned cases, increased autophagy has a negative relation to platinum response. However, some phytochemicals show anti-resistance effects *via* inducing autophagy. Zhu et al. reported that hyperoside sensitizes ovarian cancer cells to cisplatin relay on PGRMC1-dependent autophagy (Zhu et al., 2017); resveratrol can induce autophagy and apoptosis in cisplatin-resistant human oral cancer (Chang et al., 2017).

Further investigation about the changeful role of autophagy in tumor development and the complex regulatory mechanism involved in will provide more potential intervention targets and approaches for how and when to overcome platinum resistance.

## Conclusions

The resistance of platinum agents is still an ordeal. Elucidating the molecular mechanisms underlying the resistance phenomena will largely extend the clinic application of platinum agents. The main mechanisms are summarized as changed cellular platinum accumulation, increased detoxification system, increased DNA repair, decreased apoptosis, and autophagy. As the relative technologies develop rapidly, new research means such as CRISPR-Cas9, RNAi-based functional genetic screening help researchers to reexamine some key regulators and illuminate their actual impact on platinum resistance. Another notable tendency is the deeper understanding of the epigenetic modification. Non-coding RNA shows extremely complicated regulation functions on all sides and the potential to be predictive markers. The intercrossed application among genome, epigenome, transcriptome, proteome, and metabolome systems will provide a new aspect to break through the bottlenecks. But the challenges still remain as to how to utilize the emerged candidate targets as predictive markers to assign patients to more promising therapy or as therapeutic targets to improve the curative effect of platinum-based antitumor agents.

## Author Contributions

JZ, LC, and HW searched for articles, and JZ wrote the first draft of the review. JL made the figures. YK and LY revised the whole manuscript. SZ and LY supervised the process. All authors approved the submission of this manuscript.

## Funding

This work was supported by the Leading Talent of "Ten Thousand Plan" - National High-Level Talents Special Support Plan and the grants from National Natural Science Foundation of China (81773805 and 81703616)

## Conflict of Interest

The authors declare that the research was conducted in the absence of any commercial or financial relationships that could be construed as a potential conflict of interest.
